# Increased Night Temperature Negatively Affects Grain Yield, Biomass and Grain Number in Chilean Quinoa

**DOI:** 10.3389/fpls.2017.00352

**Published:** 2017-03-23

**Authors:** Jurka Lesjak, Daniel F. Calderini

**Affiliations:** ^1^Graduate School, Faculty of Agricultural Sciences, Universidad Austral de ChileValdivia, Chile; ^2^Institute of Plant Production and Protection, Universidad Austral de ChileValdivia, Chile

**Keywords:** *Chenopodium quinoa* Willd, grain yield, grain number, temperature increase, climate change, protein concentration

## Abstract

Quinoa high nutritive value increases interest worldwide, especially as a crop that could potentially feature in different cropping systems, however, climate change, particularly rising temperatures, challenges this and other crop species. Currently, only limited knowledge exists regarding the grain yield and other key traits response to higher temperatures of this crop, especially to increased night temperatures. In this context, the main objective of this study was to evaluate the effect of increased night temperature on quinoa yield, grain number, individual grain weight and processes involved in crop growth under the environmental conditions (control treatment) and night thermal increase at two phases: flowering (T1) and grain filling (T2) in southern Chile. A commercial genotype, Regalona, and a quinoa accession (Cod. BO5, N°191, grain bank from Semillas Baer, hereby referred to as Accession) were used, due to their adaptability to Southern Chilean conditions and contrasting grain yield potential, grain weight and size of plants. Temperature was increased ≈4°C above the ambient from 8 pm until 9 am the next morning. Control treatments reached a high grain yield (600 and 397 g m^-2^, i.e., Regalona and Accession). Temperature increase reduced grain yield by 31% under T1 treatment and 12% when under T2 in Regalona and 23 and 26% in Accession, respectively. Aboveground biomass was negatively affected by the thermal treatments and a positive linear association was found between grain yield and aboveground biomass across treatments. By contrast, the harvest index was unaffected either by genotype, or by thermal treatments. Grain number was significantly affected between treatments and this key trait was linearly associated with grain yield. On the other hand, grain weight showed a narrow range of variation across treatments. Additionally, leaf area index was not affected, but significant differences were found in SPAD values at the end of T1 treatment, compared to control. Little change was found in the harvest index, individual grain weight, grain protein content or water soluble carbohydrates in response to the increased night temperature in this crop.

## Introduction

Quinoa (*Chenopodium quinoa* Willd.), called “the mother grain” by the natives of the Andean highlands of Bolivia, Peru, Chile, and Ecuador (Cusack, 1984), has reached global recognition as a “superfood” for the future (Shearman, 2014). Quinoa production has almost tripled in the last two decades due largely to prices rising in response to the growing demand (FAOSTAT, 2014). This pseudo-cereal gained global attention due to its high nutritive value, associated with high protein quality (Ruales and Nair, 1992). Quinoa contains all the essential amino acids, trace elements and vitamins and is gluten-free (FAO, 2013) which makes it an excellent crop to face the challenge of increasing quantity and quality of food production globally (FAO, 2013).

Global warming is a central challenge for grain crops such as quinoa. It is expected that world average temperatures will increase between 1 and 3.7°C by the end of this century. Within these processes daily minimum temperatures are projected to increase faster than daily maximum ones (IPCC, 2014). It is noteworthy that significant increases in annual night average temperatures were recorded between 1979 and 2003 (Alexander et al., 2006), particularly in South America (IPCC, 2007; Marengo et al., 2010). Expected temperature increases in the Southern Hemisphere are foreseen for the end of spring and during the summer, from November to February (Parry et al., 2007), with the highest daily maximum temperature increase of 1–4°C (IPCC, 2014). Given that temperature is an important environmental factor affecting plant development, growth and yield (Hunt et al., 2001), it is expected that warmer climate conditions will accelerate plant development and consequently reduce the length of crop phenophases, decreasing yield (Fuhrer, 2003; Ortiz, 2008; Hatfield and Prueger, 2015) as it was demonstrated in wheat (García et al., 2015, 2016). Projections, on how and to what extent temperature increases will affect staple food crops worldwide, are crucial to future food security and cropping system adaptations. The impact of higher temperature on grain yield and associated traits has so far been assessed by simulation and field experiments for variety of crops: e.g., wheat (Asseng et al., 2009; Lizana and Calderini, 2013; García et al., 2015); sunflower (Rondanini et al., 2006; Van der Merwe, 2010); rice (Peng et al., 2004) and canola (Gan et al., 2004); however, the response of quinoa to augmented night temperatures is still unknown.

In Chile, quinoa is associated with diverse production areas as it is cultivated in a wide diversity of ecological zones. One of the two highest quinoa-yielding regions is southern Chile (Bazile, 2013), recording an average yield of 1.9 ton ha^-1^ (von Baer et al., 2009). This area (from 37 to 41°S) is also one of the most promising in the context of high yields of various crops, mainly due to favorable temperature conditions (Bustos et al., 2013; Mera et al., 2014). In the context of expected temperature increases in the Southern Hemisphere presented above, a temperature increase might therefore affect the flowering and grain filling periods of quinoa in the southern area of Chile, decreasing yield potential.

Wide genetic variability and plasticity which allow adaptation to different environments are the reasons why quinoa can grow under extreme climate conditions and poor soils (Jacobsen, 2003; Danielsen and Ames, 2004; Fuentes et al., 2009). The ability of this crop to cope with heat stress, however, has not yet been extensively researched. Mujica et al. (2001) reported that quinoa is cultivated between 15 and 20°C in the Andean environment and GxE interaction was demonstrated by Bertero et al. (2004) for quinoa grain yield and grain size through a temperature range between 9 and 22°C across three continents. It has also been suggested that Chilean sea level cultivars are less sensitive to the combination of higher temperatures and long day photoperiod, which may explain its wide adaptation (Bertero et al., 1999; Jacobsen, 2003). Nevertheless, Fuentes et al. (2012) suggested that the grain yield of quinoa in the Atacama Desert is low due to the negative effects of high-temperature stress around flowering (ranging from 31 to 35°C). To the best of our knowledge, no experimental evaluation has been performed to assess these assumptions, highlighting the need to evaluate the grain yield response of quinoa to projected temperatures.

Grain yield integrates two main components, grain number per m^2^ and average grain weight, where yield variations in grain crop species are usually associated with grain number (Peltonen-Sainio et al., 2007; Sadras, 2007; Araus et al., 2008; Gambín and Borrás, 2010). Crop resources are distributed between these two components during the grain set period resulting in a trade-off between them (Gambín and Borrás, 2010), with differences in assimilate availability per grain, i.e., a measure of the source size for the growing grains (Gambín et al., 2006). A strong positive relationship between yield and grain number has also been found in quinoa, whereas grain weight has shown a conservative behavior (Mignone and Bertero, 2007). Recently, Bertero and Ruiz (2008) reported that grain number in quinoa is highly sensitive to environmental factors from first anthesis to the end of flowering, followed by the grain filling period. Differences in grain number can also be attributed to the partitioning of biomass into the reproductive structures during the critical phase (D’Andrea et al., 2006). An improved understanding of yield responses to higher temperature during different phenological phases could uncover important clues about the sensitivity of the grain yield of quinoa to global warming.

As discussed above, yield potential of quinoa is very promising in southern Chile. Taking into account the scarcity of data on the response of this crop to environmental constrains as global warming, the objective of this study was to evaluate the impact of increased temperature on yield, grain number, grain weight and associated traits of two Chilean quinoas under the high-yielding environment of southern Chile.

## Materials and Methods

### Site Location, Experimental Design, and Plant Material

An experiment was carried out at the Experimental Agricultural Station of the Universidad Austral de Chile, in Valdivia (39° 47′S, 73° 14′W, 19 m asl) during the 2012/13 growing season. Treatments consisted of two genotypes and three thermal conditions; i.e., control, heating around anthesis (T1) and heating around grain filling (T2). These treatments were arranged in a 2 × 3 factorial combination in a Completely Randomized Design with three replications (i.e., 18 experimental units).

In order to achieve the proposed objective, a commercial variety, Regalona (Semillas Baer Temuco, Chile), was sown, as that this variety is the topmost sown quinoa in the south of Chile (virtually the only one in this area of the country). Also, an accession (Cod. BO5, N°191, grain bank from Semillas Baer), was also included in the experiment. This was done with the aim to increase our understanding of the response of the Chilean sea level genotypes to higher temperature, since this genotype is a source of germplasm for the quinoa breeding program. In addition, Regalona has presented a higher grain weight than Accession but similar phenology in previous evaluations. Thermal treatments consisted of: control at ambient temperature, and two independent increased temperature conditions, i.e., at 12 days after the first anthesis (T1) and at 12 days after the end of flowering, i.e., during the grain filling period (T2) (Supplementary Figure A1). Both treatments were applied for a 12-day period as in previous study of wheat in southern Chile (Lizana and Calderini, 2013). Temperature was increased at night, by approximately 4°C above the ambient.

### Sowing and Management Conditions

The experiment was sown on October 30, 2012 in a Typic Hapludand soil, developed from volcanic ashes, pH 5.8 and organic matter by 15% (for more details see Valle et al., 2009; Dörner et al., 2015). Seedling emergence was recorded on November 13, 2012. The experimental units (plots), oriented north–south, were 2.8 m long and 4.2 m wide, 35 cm between rows (11 rows per plot) and 20 cm between plants, with 13 plants per row at a plant rate of 12.1 plants m^-2^. To avoid aluminum and biotic constrains, 4kg m^-2^ of CaO and 50g m^-2^ of fumigant (DAZOMET) were applied in the soil before sowing. Plots were kept free of fungal diseases and insect pests through the entire growing season as recommended by Semillas Baer Co. (Chile). Weeds were removed by hand. To avoid water stress, plots were regularly watered throughout the growing season to complement seasonal rainfall. Experimental units were fertilized before sowing with 20 kg P ha^-1^ and 18 kg N ha^-1^ and two fertilizations with urea totaling 200 kg N ha^-1^, were applied at 30 and 60 days after sowing.

The thermal treatment was performed using transparent polyethylene (100 μm thick) chambers, of 2.8 × 2.8 and 1.5 m (length, width, and height) equipped with electric heaters controlled by thermostats as in prior experiments (Savin et al., 1996; Ugarte et al., 2007; Lizana and Calderini, 2013). The tops of the chambers were set at night (8 pm) and removed in the morning (9 am). This was done to increase the night temperature only, avoiding a daily maximum temperature above 30°C (heat shock) in the chambers and to prevent reductions in solar radiation by the polyethylene film. Heating was controlled by thermal sensors placed at panicle height, connected to a temperature regulator as in Lizana and Calderini (2013). Temperature was recorded, both in the chambers and outside, by data loggers (Cavadevices, Buenos Aires, Argentina). Mean air temperature in the field was recorded by the weather station set (Davis Vantage Pro, USA) positioned 50 m from the experiment. Average day temperature during the treatments was calculated by using start temperature of the treatments (from 8 pm until 9 am the next morning) and ambient temperatures.

### Measurements

#### Development and Biomass

Developmental stages were recorded as: emergence, visible flower bud, first anthesis (at least one flower open on 50% of the plants in the main inflorescence from each plot), end of anthesis (no more flowers open on at least 50% of the plants from each plot), and physiological maturity.

These stages also defined four developmental phases: vegetative, reproductive, flowering and grain filling (Bertero and Ruiz, 2008). Grain filling duration was calculated by subtracting the days up to anthesis from the time to physiological maturity.

Biomass was sampled at each plot by harvesting five contiguous plants in central rows, not including border plants. In treatment T1, samplings were made at: (i) 14 days after visible floral bud, (ii) the beginning of the heating treatment (12 days after first anthesis), (iii) 6 days after starting the treatment, (iv) the end of the treatment, (v) 23 days after the treatment and (vi) physiological maturity for both genotypes. Therefore, samples for the T1 treatment were obtained at 59 (the beginning of the treatment), 65, 68, 71, 83, 94, 100, and 128 (physiological maturity) days after emergence (DAE). In the T2 treatment, samplings were made at (i) 14 days after visible floral bud, (ii) the end of anthesis (beginning of the grain filling period), (iii) 12 days after the beginning of the grain filling period (beginning of the treatment), (iv) 6 days after sampling iii, (v) the end of the treatment, and (vi) physiological maturity. Thus in this treatment samples were harvested at 76 (the beginning of grain filling), 88 (beginning of the treatment), 91, 94, 97, 100, 115, and 128 DAE. Plants from the corresponding control plots were similarly sampled. At harvest maturity, 10 contiguous plants were sampled per plot.

Plant biomass was divided into: green leaves, senescent leaves, stem (main stem and branches) and inflorescences (main stem and branches) when present. To calculate leaf area index (LAI), leaf area of green leaves was measured by an electronic leaf area meter (LI 3100, Licor Inc., Lincoln, NE, USA) and accordingly calculated. Grain weight was measured in each plant; from which 3 × 50 grains were taken from the middle position of the panicle. Grains were sampled with petal leaves to weigh fresh biomass. Once dried in an air-forced drying oven at 60°C for 72 h, grains were weighed to record dry biomass. Grain number per m^-2^ was calculated as the ratio between grain yield and average grain weight at harvest of each plot. Aboveground biomass was calculated as the sum of all organs. Grain yield of the main stem and branches were determined at the end of the growth cycle separately, whereas total yield was considered their sum. The harvest index (HI) of each experimental unit was calculated as the ratio between grain yield and aboveground biomass at maturity. Crop growth rate (CGR) was calculated as the slope of the linear regression between biomass and time (in days).

#### Chlorophyll Content

A SPAD portable chlorophyll meter (SPAD-502 leaf chlorophyll meter, Minolta Corporation, Osaka, Japan) was used to measure the light transmission spectrum of chlorophyll a and b at two leaf positions: (i) the top third and (ii) from the middle third of the main stem, at each evaluation (five plants per plot) in thermal treatment. The measured values (arbitrary units) showed a linear relationship with total chlorophyll content in quinoa (Bertero, 2001).

#### Water-Soluble Carbohydrates (WSC)

Water-soluble carbohydrates (glucose, fructose, and sucrose) were extracted from the main stem and branches at the beginning and end of the thermal treatments. Five plants per plot were sampled for each evaluation. WSC was measured according to the Analysis of Agricultural Materials (A.O.A.C., 1996. Official Method; Methods 14, Carbohydrates, Soluble, in Herbage).

#### Grain Protein Content

Grain protein concentration (%) at harvest maturity was measured from the bulk grain at each replicate, using the Kjeldhal method and a conversion factor of 6.25, i.e., equivalent to 0.16 g nitrogen per gram of protein (Merrill and Watt, 1973).

### Data Analysis

The effects of increased temperature treatments were analyzed using a factorial analysis of variance (ANOVA) with two genotypes and three temperature regimes. An ANOVA was performed using the STATGRAPHICS^®^ Centurion XV software, (StatPoint Inc., 2005). Multiple means were compared with Fisher’s Least Significant Difference (LSD) test, where the probability level of *p* ≤ 0.05 was considered statistically significant. Regression analyses were used to measure the association between variables.

## Results

### Environmental Conditions and Crop Phenology

Environmental conditions, other than temperature during the thermal treatments, were similar for both genotypes across the growing season. Thermal treatments increased the daily average temperature in the chambers by 2.1°C above the ambient temperature for the 12 days of treatment across genotypes and phenophases (**Table 1** and Supplementary Figure A1). With regard to night temperatures (between 8 pm and 9 am), heating increased temperature in the chambers by 4.3°C (T1) and 4.2°C (T2), across the 12 days treatment in Regalona, and by 3.8°C (T1) and 3.7°C (T2) in Accession (**Table 1** and Supplementary Figure A1).

**Table 1:** Mean temperature of control (from emergence to physiological maturity at ambient temperature) and increased temperature (T1 and T2) treatments in Regalona and Accession.

Genotype	Timing of temperature increase	Mean temperature during the 12 day treatment (from 0 am to 12 pm;°C)		Mean temperature during the 12 day treatment (from 8 pm to 9 am; °C)	
		Control ambient	T1	Control ambient	T1
Regalona	Flowering	20.5°C	22.8°C**^∗^**	17.9°C	22.2°C
Accession		18.5°C	20.6°C**^∗^**	16.4°C	20.2°C

		**Control ambient**	**T2**	**Control ambient**	**T2**

Regalona	Grain filling	16.1°C	18.2°C**^∗^**	14.9°C	19.1°C
Accession		15.6°C	17.6°C**^∗^**	13.6°C	17.3°C

*^∗^Average day temperature was calculated from the beginning 8 pm to the end 9 am of heating under both T1 and T2.*

**FIGURE 1:**
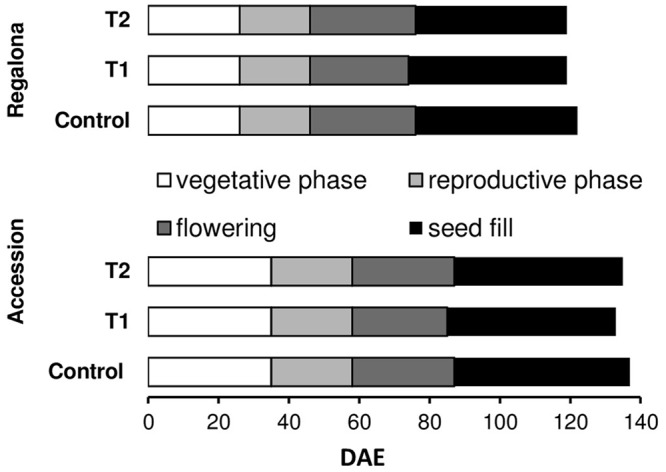
**Phenological phases duration from emergence in control and increased temperature treatments (T1 and T2) of Regalona and Accession.** DAE, days after emergence.

The average temperature for cv. Regalona during the crop cycle (from emergence to physiological maturity) was 15.9°C at the control treatment, and 16.1°C under thermal increase (under both T1 and T2). The average temperature for Accession between emergence and maturity was 15.6°C in the control plants, 15.9°C under T1 and 15.8°C under the T2 treatments (Supplementary Figure A1). Average incident PAR, between emergence and physiological maturity, was 11 MJ m^-2^ day^-1^ for Regalona and 10 MJ m^-2^ day^-1^ for Accession (Supplementary Figure A1). The crop cycle was divided into four phases: vegetative, reproductive, flowering and grain filling, following Bertero and Ruiz (2008). Regalona reached visible flower at 25 days after emergence, while Accession showed a longer vegetative phase, i.e., 35 days across treatments (**Figure 1**). Increased temperature negligibly shortened the growing cycle in both genotypes with no significant difference (*P* > 0.05). Regalona growth cycle was shortened by 3 days under both temperature treatments (T1, T2), while Accession accelerated the growth cycle by 4 days under T1 and by 2 days under T2 (**Figure 1**).

### Effect of Increased Temperature Treatments on Grain Yield, Aboveground Biomass, and Yield Components at Harvest

**FIGURE 2:**
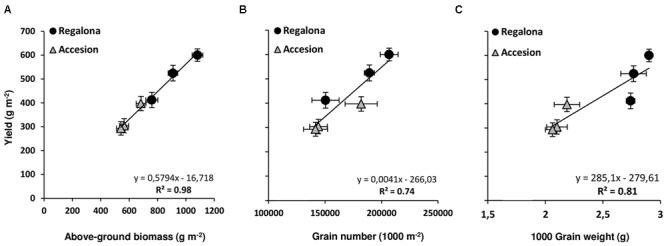
**Relationship between grain yield and above-ground biomass (A)**, grain number **(B)**, and plant height **(C)**. Bars show the standard error (SE) of the means.

Grain yield was affected (*P* < 0.001) by both genotype and thermal treatments. In the controls, Regalona exceeded Accession yield by 51% (**Table 2**). Increased temperature at flowering reduced grain yield by 31% in Regalona and 23% in Accession. When temperature was increased during grain filling, grain yield of Regalona was not significantly affected by heating, though reduced by 13%, while Accession showed similar sensitivity (-26%) to that at flowering (**Table 2**). Like grain yield, aboveground biomass was also affected (*P* < 0.001) by genotype and thermal treatments. The sensitivity of biomass to increased temperature at flowering (T1) was higher in Regalona (29%) than in Accession (17%), while during grain filling (T2) biomass decreased by 15 and 21% in Regalona and Accession, respectively (**Table 2**). A positive linear association was found between grain yield and aboveground biomass across treatments when they were plotted together (**Figure 2**). By contrast, the HI was not affected by either genotype or increased temperature treatments (**Table 2**), and no association was found between grain yield and HI. Plant height differed between genotype and treatment (*p* < 0.05), where the shorter plants were found in the more productive cultivar, Regalona (**Table 2**).

**Table 2:** Yield and yield components at harvest of Regalona and Accession under temperature treatments (Control: C; Anthesis: T1 and Grain Filling: T2).

Genotype	Treatment	Timing of treatment	Grain yield (g m^-2^)	Above-ground biomass (g m^-2^)	Harvest index	Plant height	GN grains (m^-2^)	GW (mg)	Protein (g 100 g^-1^ DM)
Regalona	C	Flowering	600.1	1081.3	0.55	70.4	206769	2.90	16.8
	T1	Grain filling period	412.1	759.8	0.57	56.9	150400	2.74	17.1
	T2		524.4	909.0	0.57	63.7	189210	2.76	17.7

Accession	C	Flowering	397.0	683.7	0.58	82.5	181964	2.18	17.6
	T1	Grain filling period	303.9	564.8	0.54	84.7	144150	2.10	18.0
	T2		292.8	538.1	0.54	72.8	141558	2.06	18.2

Mean			421.7	756.8	0.56	71.8	169008	2,46	17,6
SE			28.7	47.7	0.0076	2.5	6946	0.08	0.175
G			^∗∗∗^	^∗∗∗^	NS	^∗∗∗^	^∗∗^	^∗∗∗^	^∗^
HT			^∗∗∗^	^∗∗∗^	NS	^∗^	^∗∗^	NS	NS
GxT			NS	^∗∗^	NS	^∗∗^	NS	NS	NS

*Values are means of tree replicates. Asterisks indicate significant differences between treatments at ^∗^*P* < 0.05, ^∗∗^*P* < 0.01, and ^∗∗∗^*P* < 0.001, while NS, not significant (*P* > 0.05) with Fisher’s least significant-difference (LSD) test. DM, dry mass; Protein, Nx6.25; G, genotype; T, treatment, HT, heating treatment.*

With regard to yield components, significant differences were found in grain number per square meter between genotypes and among thermal treatments (**Table 2**). In Regalona, grain number was significantly (*P* < 0.05) affected by high temperatures only under T1 (27% reduction); however, in Accession grain number was sensitive to heating under both T1 (20%) and T2 (22%). Interestingly, a positive association (*P* = 0.01) was found between grain yield and grain number across the treatments (**Figure 2**).

As expected, grain weight significantly differed between the genotypes (**Table 2**). Despite grain weight being reduced by 5 and 4% due to the thermal treatments in flowering and grain filling, these reductions were not significant (**Table 2**). In addition, no interaction between genotype x thermal treatments was observed for this trait. A linear relationship was found between yield and grain weight (**Figure 2**), but this association was mainly due to differences between genotypes. Therefore, changes in grain yield were explained by grain number.

### Biomass Partitioning in Main Stems and Branches

**FIGURE 3:**
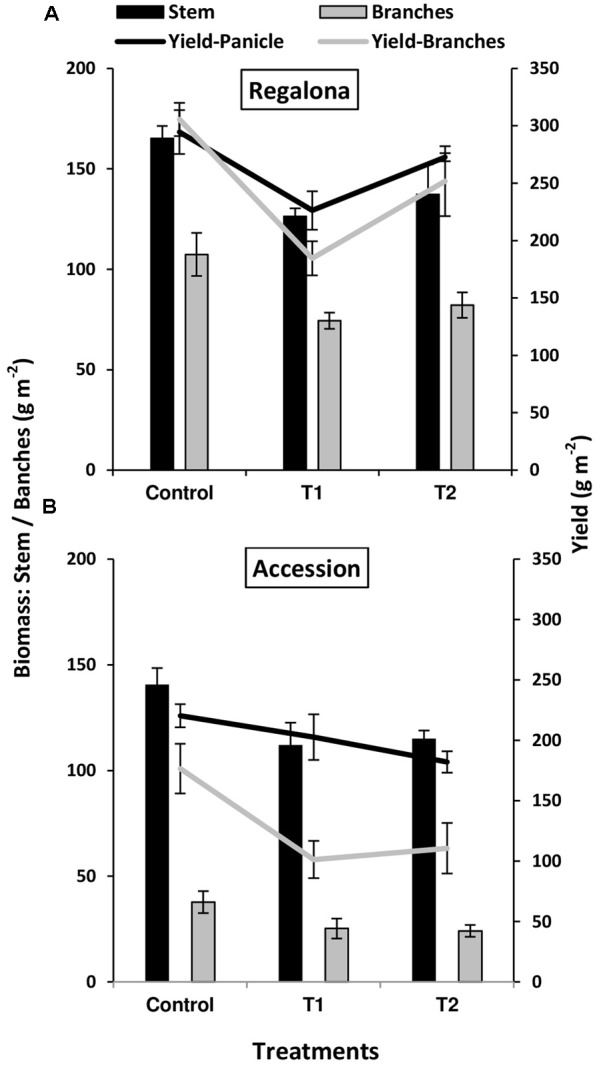
**Grain yield (lines) and biomass (bars) of main stem (black) and branches (gray) of control and heating treatments (T1 and T2) in Regalona (A)**, Accession **(B)**. Bars show the SE of the means.

A complementary analysis was made by assessing the contributions of main stems and branches to grain yield and aboveground biomass across the treatments. In Regalona, panicles from the main stems and branches of the control plants contributed almost the same to grain yield, i.e., 294.5 and 305.5 g m^-2^, respectively (**Figure 3**). Main stems and branches of Accession showed a greater contrast than in Regalona for both grain yield and biomass partitioning in the control plants. Main stems of Accession over-yielded branches in both grain yield and aboveground biomass, but particularly in the latter (**Figure 3**).

Increased temperature treatments significantly (*P* < 0.05) affected the grain yield of main stems, but not biomass (*P* > 0.05; **Figure 3**) in Regalona. Both traits were decreased by 23% under T1 and by 16 and 7% under T2, respectively. Grain yield and biomass of branches were both significantly affected (*P* < 0.05) by the thermal treatment, showing a linear relationship between them (*R*^2^ = 0.50; *P* = 0.03). Grain yield of branches was decreased by 38% under T1, and 17% under T2, while biomass of branches decreased by 30% under T1 and 23% under T2, compared to the controls. Grain yield and biomass of the main stems were not affected in Accession and no association was found between these traits (**Figure 3**). The difference in main stem grain yield was -8% than control under T1 and -17% under T2, while biomass decreased by -20% under T1 and by -8% under T2. No difference was found in the biomass of branches, while grain yield was affected (*P* < 0.05) and a positive association (*R*^2^= 0.85; *P* < 0.001) was observed between them. Additionally, the biomass of branches decreased by 30 and 32% under T1 and T2, respectively, while grain yield was more affected and decreased by 42% under T1 and 37% under T2.

### Aboveground Biomass Accumulation under Thermal Treatments

**FIGURE 4:**
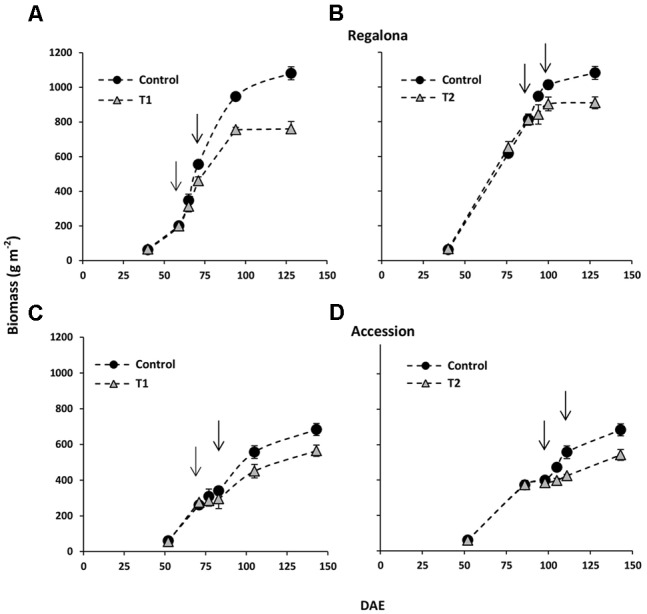
**Time-course of above-ground biomass offor control and T1 (A)** and T2 **(B)** treatments in cv. Regalona **(A,B)** and Accession **(C,D)** days after emergence (DAE). Arrows indicate the beginning and the end of heat treatments. Bars show the SE of the means.

Taking into account the association between grain yield and biomass shown in **Figure 4A**, and to obtain data on the sensitivity of biomass production at higher temperatures in quinoa, we also evaluated the time-course of aboveground biomass was evaluated in the present study. At the beginning of T1, the aboveground biomass was similar (*P* > 0.05) between the control plots and thermal treatments in Regalona and Accession (**Figure 4**), however, at the end of heating, i.e., 12 days after the beginning of T1, lower (*P* < 0.05) biomass was recorded in both genotypes (-17 and -13% for Regalona and Accession). Differences in biomass between T1 and the control treatments were increasing until harvest (**Figure 4**), when the control treatment showed significantly higher biomass (29%; *P* < 0.01) than T1 in Regalona and Accession (17%; *P* < 0.05) supporting the key hypothesis on the sensitivity of the biomass to higher temperature (**Table 2**).

As under T1, aboveground biomass under T2 was similar to the control treatment at the beginning of the treatment in Regalona and Accession (**Figure 4**) and significantly lower biomass was found at the end of heating in Regalona (-16%) and Accession (-20%; *P* < 0.05) (**Figure 4**).

**FIGURE 5:**
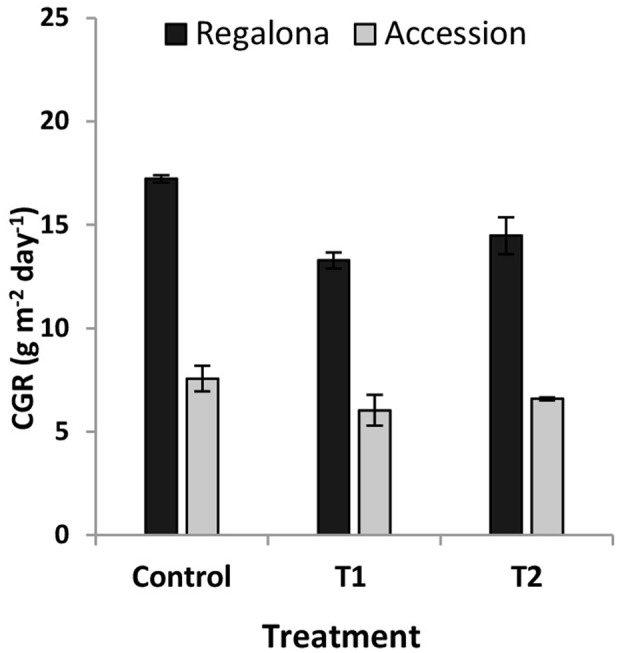
**Crop growth rate (CGR) of cv. Regalona and Accession (a) under control, temperature treatments (T1 and T2) calculated for entire crop cycle**.

**FIGURE 6:**
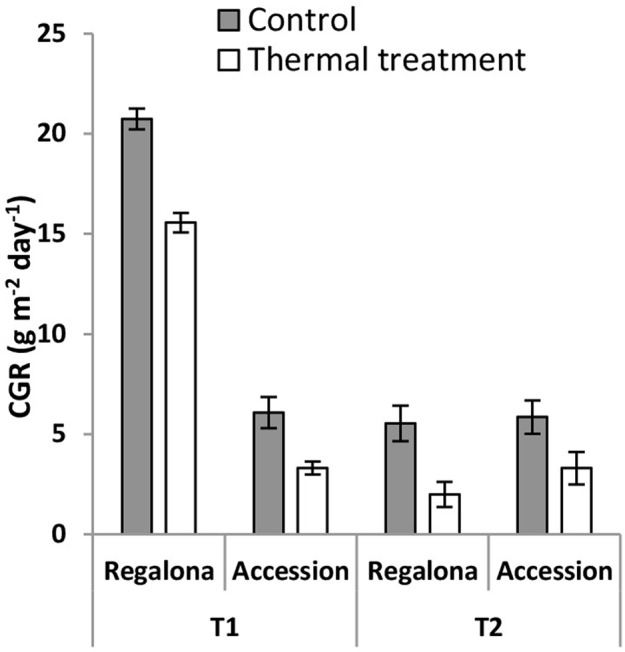
**Crop growth rate of cv. Regalona and Accession under control, temperature treatments (T1 and T2) calculated for the 12 days thermal treatment period.** Bars indicate SE of the means.

The CGR calculated from 50 DAE to maturity was significantly (*P* = 0.007) affected by the thermal treatments (**Figure 5**), whereas the effect of heating on the whole crop cycle duration, i.e., from emergence to maturity, was negligible (**Figure 1**). Heating decreased CGR by 23% under T1 and 6% under T2 for Regalona during the period from emergence to maturity, while this trait was not affected in Accession. Calculating CGR only during the heating treatment, i.e., 12 day-period, this rate decreased by 25% (*P* = 0.001) under T1 and by 63% (*P* = 0.03) under T2 for Regalona (**Figure 6**). In Accession, CGR was significantly decreased during T1, i.e., 45% (*P* = 0.03) and similarly decreased (43%) under T2, though not significant.

**FIGURE 7:**
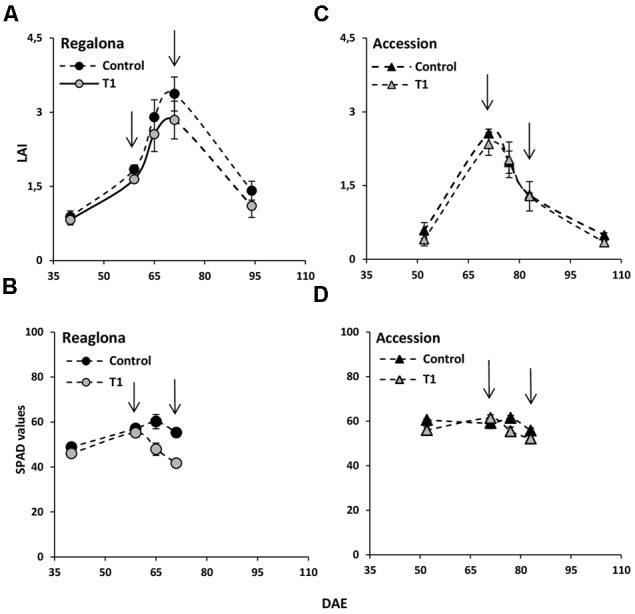
**Time-course of LAI (leaf area index) and SPAD values under T1 treatment in cv. Regalona (A,B)** and Accession **(C,D)** in days after emergence. Bars show the SE of the means.

Leaf area index and chlorophyll concentration (estimated by the SPAD values) were also measured. LAI was not affected under the T1 treatment, neither in Regalona or Accession (**Figure 7**). However, SPAD values were lower in both genotypes (*P* = 0.01 for Regalona and *P* = 0.04 for Accession), compared to control. LAI values were clearly lower under T2 (*P* = 0.01). At the end of T2, LAI reached 56% reduction compared to the control, mainly due to leaf senescence, which begins at the end of flowering (beginning of the grain filling period).

### Water-Soluble Carbohydrates (WSC)

As heating might affect WSC, which contributes to the size of the source for growing grains, this trait was measured by individual stem category. Similar WSC concentration was found at the beginning control and T1 treatment plots of each genotype (data not shown). Immediately after heating, the evaluation of WSC showed no differences in the WSC concentration in either main stems (*P* = 0.55) or branches (*P* = 0.47) between the control treatment (1.3 and 0.7 g/plant in main stem and branches, respectively) and T1 (1.0 and 0.6, respectively) treatment in Regalona. Similar results were found in Accession, where higher temperatures had no effect on either main stems (*P* = 0.72; 1.7 and 1.3 g/plant in main stems and branches, respectively) or branches (*P* = 0.20; 0.2 and 0.16 g/plant). Similar to T1, the WSC concentration was not affected (*P* > 0.05) by T2 in the main stems or branches between the control and thermal treatments in either Regalona or Accession (data no shown).

### Grain Protein Concentration

Genotypes showed differences in grain protein concentration, where Accession reached higher values (**Table 2**). The higher temperature did not affect the grain protein concentration (**Table 2**). A positive association was found between the protein concentration and grain weight (*P* = 0.03), whereas no association (*P* = 0.07) between protein concentration and grain yield was found. However, it is important to highlight that the protein content-grain weight relationship was mostly caused by genotype differences in the protein concentration.

## Discussion

This study evaluated the effect of increased night temperature on yield and associated traits of quinoa and addressed the gap in literature on the response of quinoa to elevated temperatures, particularly for Chilean sea level cultivars.

Increased temperature treatments were chosen in this study considering to the future climate change scenarios forecasted for southern Chile (Department of Geophysics, University of Chile, 2006; IPCC, 2014). The heated treatments consisted on ≈4°C above the ambient temperature at night for a period of 12 days, which accounted for around 2°C increase above the 24 h ambient air temperatures (**Table 1**). The main findings show that grain yield of quinoa is highly sensitive to increased night temperatures, especially during flowering. Taking into account this original data and its potential relevance for future research, we also compared the results were compared to evaluations carried out on other crops.

Increased temperature had a negative effect on grain yield which was explained by the adverse impact of high temperature on biomass production, what is in line with previous studies evaluating wheat (Calderini et al., 1999; Peltonen-Sainio et al., 2007; Gambín and Borrás, 2010; Sadras and Slafer, 2012). It has also been shown that chickpea is highly susceptible to heat stress during flowering and grain filling due to the negative effect of temperature on biomass production (Wang et al., 2006; Devasirvatham et al., 2012). Interestingly, the HI was insensitive (*p* > 0.05) to the thermal treatments in our study, even when plants were heated during grain filling.

The negative impact of high temperature on the crop biomass of quinoa could be explained by the effect on either the crop cycle duration or CGR. A great deal of evidence has shown that the rise of temperature increases the development rate which, in turn, reduces biomass production and grain yield (e.g., Fischer and Maurer, 1976). For example, previous experiments of wheat have shown that higher temperatures increase the rate of development, reducing crop radiation interception with concomitant reductions in dry matter accumulation and wheat grain yield (Farooq et al., 2011; García et al., 2015). This response is different from our study on quinoa, where only CGR was affected by temperature increase and there was no impact on the crop cycle. However, given the genetic variability shown by different traits in this specie (De Santis et al., 2016), we cannot discard genetic variability of CGR and the development rate to increased temperature in quinoa. The biggest impact found in this study was in the biomass of branches; which was considerably smaller in the main stems. As LAI was not significantly affected at the end of the thermal treatment under T1, it may be safe to suggest that heating affected the photosynthesis capacity of the plants. This would also be true for T2, as the senescence process was accelerated by the thermal treatment.

We also found that grain yield was more closely associated with grain number (*R*^2^ = 0.92; *P* = 0.001) than grain weight in this experiment, which is consistent with previous studies on quinoa (Bertero and Ruiz, 2008; Gómez et al., 2011) and other grain crops (Peltonen-Sainio et al., 2007; Sadras, 2007; Gambín and Borrás, 2010; Sadras and Slafer, 2012). Several studies have reported that grain yield is reduced due to higher temperatures at flowering by decreasing grain set in crop species like chickpea (Wang et al., 2006), canola (Morrison and Stewart, 2002), buckwheat (Michiyama and Sakurai, 1999; Michiyama et al., 2001), soybean (Jiang and Egli, 1995), and temperate cereals (Calderini et al., 1999; Wheeler et al., 2000; Ugarte et al., 2007; Lizana and Calderini, 2013; García et al., 2015). Regalona and Accession were highly sensitive to elevated temperatures during flowering, adjusting grain set (from main stem and branch panicles). As suggested by Bertero and Ruiz (2008), the efficiency of quinoa grain setting can be affected during grain filling, altering the final grain number by grain abortion. In soybean, abortion and abscission can occur even in immature pods, with the latter also being the most vulnerable (McBlain and Hume, 1981); by contrast, immature grains are more likely to be aborted than larger grains (Van Steveninck, 1959; Abernathy et al., 1977; Westgate and Peterson, 1993). This can be caused by different environmental stresses such as high temperatures (Duthion and Pigeaire, 1991; Nilsen and Orcutt, 1996). In addition, the quinoa commercial supply chain in Europe and North America recommends sowing quinoa in a mild climate, such as in the Loire Valley in France and the northern part of Minnesota, where summer temperatures do not exceed 23°C on average (Abbottagra, 2014; SFA, 2014). Furthermore, average day temperatures in Valdivia from November to March (almost the entire growing season) are exceedingly lower than in the Loire Valley from May to September. In the present study, the average day temperature was below 23°C even in thermal treatments, but both biomass and grain yield were negatively impacted. This suggests that higher night temperatures (by approximately ≈4°C above the ambient temperature) for a short period will negatively affect these traits. On the other hand, the temperature increase treatments had no effect on grain protein concentration.

The grain weight of quinoa was highly conservative compared to grain number in this study, which is in line with other studies (Mignone and Bertero, 2007; Bertero and Ruiz, 2008; Lopez, 2008; Spehar and Rocha, 2009; Gómez et al., 2011). Therefore, quinoa shows sensitivity to elevated temperatures only through an adjustment of the grain number, perhaps as an avoidance mechanism or as a strategy for tolerating this abiotic stress (Jacobsen et al., 2009). Nevertheless, significant changes were observed in quinoa grain weight by Munir (2011) and Gómez et al. (2013) in experiments evaluating sowing dates in contrasting environments (Faisalabad, Pakistan and Buenos Aires, Argentina).

The sensitivity of quinoa grain yield to temperature during flowering found in the present study (a reduction of 31.3% in Regalona and 23.5% in Accession) is consistent with previous manipulations of the source-sink ratio around this phenological stage (Mignone and Bertero, 2007; Bertero and Ruiz, 2008). Therefore, the importance of biomass accumulation during flowering is a key for grain number determination in quinoa.

## Conclusion

Grain yield of the most sown cultivar of quinoa in southern Chile (Regalona) and a source of germplasm like Accession were highly sensitive to increased night temperature around flowering; however, this constraint had a different impact at grain filling. Thermal treatments (by approximately ≈4°C above the ambient temperature) affected biomass production and grain number only when applied during flowering in Regalona and when applied either at flowering and grain filling in Accession. In addition, grain yield was closely associated with aboveground biomass and CGR, and there was little change in the HI across treatments. Individual grain weight and grain protein content were not sensitive to either the T1 or T2 treatments.

## Author Contributions

JL proposed the experiment, carried out the measurements and analyzed the data. DC proposed the hypothesis, collaborated with JL in planning the experiment and measurement and collaborated in the writing of the manuscript.

## Conflict of Interest Statement

The authors declare that the research was conducted in the absence of any commercial or financial relationships that could be construed as a potential conflict of interest.
