# Supplementary figures and images for: Increased Night Temperature Negatively Affects Grain Yield, Biomass and Grain Number in Chilean Quinoa
Source: Front Plant Sci. 2017 Mar 23;8:352. doi: 10.3389/fpls.2017.00352 (PMC5362734; doi:10.3389/fpls.2017.00352)

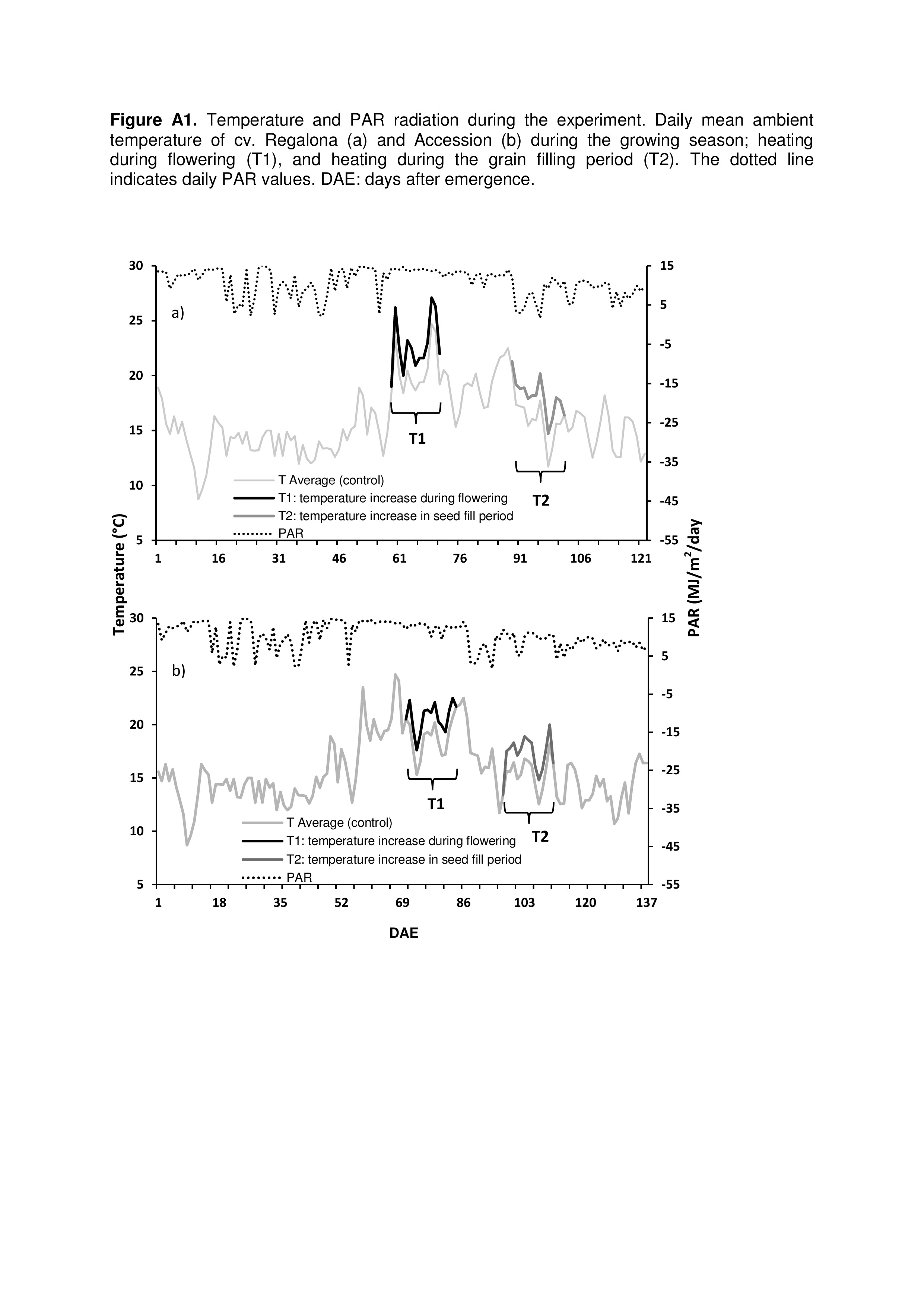

Supplement: Supplementary file 1 [file Image_1.JPEG]
